# Are You a Reading Man?

**Published:** 1865-12

**Authors:** L. D. Shepard

**Affiliations:** Salem, Mass


### ARE YOU A READING MAN?

BY L. D. SHEPARD, D. D. S., SALEM, MASS.

[Read before the Massachusetts Dental Society, at Boston, Oct. 2d, 1865.]

During the past two years, more than ever before, my attention has been directed to the subject of professional education.

Within this period we have witnessed great progress, much of which is undoubtedly due to the formation of so many local societies.

It is impossible for men to meet, month after month in these gymnasiums of thought, without acquiring new vigor and acumen. One ot the first impressions that the new member receives, is a consciousness that the field of research is broader than he supposed, and that he himself has only crossed its border. He begins to study and investigate, and a few months works a decided change in his views of the profession. That this is the natural result of associative effort, is obvious. I will only mention as an illustration, that during the past winter, three members of the Connecticut Valley Association, who had been in practice five, eight and nine years respectively, closed their offices, attended lectures at one of our dental colleges, and took their degrees. One of them remarked to me, a few months since, that he had never ceased to regard it as the most important event of his professional life, that he was induced to attend our meeting at

Greenfield, Mass., in January, 1864. His progress since that time has been rapid, and he attributes it all to the inspiration caught at our meetings. The same is true of the other two, and in fact, of all the members. I have cited this case as an illustration of the results which such associations should exhibit everywhere. It is not an isolated case, and yet, to me, is one of great force, as it comes from within the circle of my intimate knowledge, and is a result, to a certain extent, of my own earnest labors. This society, to which I have transferred a large share of my interest, undoubtedly displays to you all, the same reviving spirit, as a direct result of its organization. The same is true all over the land, and yet we feel, but a few sheaves have been gathered, while the great field remains unharvested. Maine and Rhode Island, as yet, have no societies; has New Hampshire, or Vermont? Although, these two States have large representations in the Connecticut Valley and Merrimack Valley Association, I can conceive of no reason why these States should be so far behind other states, except it be that no one has taken the iniatory steps. Experience has shown, that all that is needed is for some active and judicious man to make the first move, and others will follow.

But, I had another object in preparing this article, and have permitted the preceding remarks to open the subject, as they are so intimately connected with it. In fact, it is impossible to speak of one division of professional education without touching upon all. Our development must be *symmetrical*, *skill* must keep pace with *science*, and *integrity preside over all.*

There is one direction in which, I fear, our societies have failed to produce their proper results. Our meetings ought not only make us better operators, and better informed, from the observations and remarks thrown out, from month to month, but awaken in each mind, a thirst for knowledge, and a desire for *personal* investigation, during our leisure hours. Has this result been gained?

To answer this question, we must inquire in what way this study must be prosecuted, and what means must be seized upon, to gain this increase of knowledge.

Individual investigation is one of the most important. It must be a "habit" with us to make each case a study, particularly, if any doubt of the result exists. To do this, we must keep a case book, in which the history shall be written, of the first, and each subsequent examination, with the course of treatment, and reasons for the same. In this way we can learn in time, *why we failed.* The use of the microscope, is another. I would like to know, how many microscopes are used in this society, and how many of our number have never seen the Haversian canals, lacunæ, canaliculi, etc., of the dentine, the circulation of the blood, and the almost endless objects of interest which this wonderful instrument unfolds to view. But, the easiest and most available means of keeping our minds active, and enlarging the sphere of our information, is the *scientific reading* of the day. To this point, I desire to call your *particular attention.* I ask, seriously, as a profession, do we *read* enough? Are we up to the times, as a learned profession, in the great throes of thought, science, and progress, which agitate the world? How many of us can converse on the great problems of the day, throughout the world, with anything more than the shallow commonplaces of ordinary life? Is this enough for a learned profession? Can the same be said of the mass of the clergy, the law, and regular medicine? We will grant, for arguments sake, that this general erudition is not necessary for our daily duties. Furthermore, we will say, that it is pardonable in a man whose early advantages were not great, and whose time is occupied in a laborious profession, if he is not thoroughly posted on general topics. On this point we may, perhaps, all agree. But where this same ignorance, and neglect to attempt its removal, is the general status of the profession on professional topics, are we not equally agreed to plead guilty, very guilty?

Is there any excuse for our conduct, if this is the state of the profession to-day? Our scientific text-books are not numerous. How many of us have read them all? They contain errors, and many ideas behind the times, yet their general principles are correct, and absolutely necessary for intelligent practice. But are there no recent publications where we can find the new theories, with the exposure of errors, old and new? If so, can we turn our backs on these, and claim to be educated men? The one grand curse upon our profession, from its beginning, to the present day, has been the lamentable and very general ignorance of its members. Surely, in this day of light, and progress, and general information, where the grand christian doctrine, that "it is more blessed to give than to receive," is the rallying cry of every profession, and knowledge is almost as free as the air we breath, what excuse can we render at the bar of public opinion, if we, as a profession are still groping our way in the dark ages?

A moment ago, I spoke of the necessity of familiarity with the old text-books, and the recent publications of the profession. Of course you all understand, by the latter, I refer to the dental magazines. You know what they are, their standing, and the style of their articles. I desire to plead earnestly for their more extensive circulation. Foremost stand the Dental Cosmos, and Register of the West, both able magazines and worthy of our patronage. From month to month, I welcome their familiar faces. I take others, but regard these as best adapted for general circulation. There are a number of publications issued as a means of advertising certain institutions, patents and manufactories, which have for the most part a gratuitous circulation. Some of these contain matter of interest and value, but for obvious reasons need no plea of mine.

Some may think that these magazines (Dental Cosmos and Register) do no[illegible] come up to their standard, or are not conducted as such magazines should be. Who, I ask, but those very men are to blame for this? Is it any excuse for them to stand aloof, because that which is, does not exactly accord with their notions? Or rather, are they not guilty, if, having decided ideas, they keep them to themselves, and thus deprive the profession of their good counsels?

I regard the magazines as exponents of the profession, conducted by men of foresight and good judgment, as well as of liberal and elevated views; they consult what is required by the profession, and publish what pieces of decent merit the profession at large furnish them with. Can we, entertaining such views, look with favor on men of talent, who hide their talent in a napkin, and keep it from the world? Can such condemn a magazine, as not what it should be, when they have made no effort to change its tone? I know of no one among you who entertains such feelings as these; but I know of some who do, and whose course I must condemn.

Another reason why every dentist should be a subscriber to one magazine, at least, (more if he has the means) is the duty he owes to the profession, to contribute all he can to its elevation. If scientific publications are valuable — who doubts it at this day? they must be sustained, and every lover of his profession must be an agent to elevate their standard, and increase their circulation. "Why stand we here idle?"

I have spoken of our individual responsibility for the standing of the current literature of the profession, and of our duty to the profession, to help to sustain it, even if we do not read the magazines, or need their instructions. I now come to the practical question, whether duty to ourselves permits us to ignore them. What think you of a family where the newspaper does not make its daily or weekly visit? Is such generally regarded as intelligent? Do you regard them as fit companions with whom to pass a social evening? On the contrary are they not always ignorant, superstitious and narrow-minded? *In what differs an office* without a professional paper? I do not see how any operator can keep up with the times, and have his mind bright and active, unless he reads every month some professional writings which are fresh and practical. How many of you agree with me in theory? You may read things which are contrary to your experience. There is a chance that you may be in error, that your observations may not have been as carefully made as those of others. Or the writer may be wrong, and do vast injury unless you correct the mistake. How many opportunities of good are lost, if you are remiss in your duty in this respect, can never be known. "Let your light so shine, that men may see your good works."

Does it not seem to you to be almost needless to argue this question? And do you think I am over-excited, and spending my breath for naught? I tell you, brothers, I know what I am talking about, and it is with sadness, I am compelled to speak thus earnestly.

To any one of you who takes none of the literature of the profession, let my words be addressed personally, and received kindly, as becomes brethren engaged in a common cause, and inspired by a noble spirit.

You have no idea of the necessity of this appeal. Each one of you knows personally about himself. But, I know how many copies are taken in New England, and who are the favored ones. You would be surprised, if I should tell you how few, how very few, are taken in this city, and yet this city is not behind other portions of the land.

My position on the Committee on Dental Education of the American Dental Association has led me to study this subject with more than ordinary interest, and has furnished superior facilities.

Some of you may be aware of my interest in this subject. All, I trust, will give me credit for an honorable motive in making this appeal.

Well rewarded shall I be, if I can contribute in any way, and at any time, to elevate our noble profession, and place it, higher still, on the rolls of the noble and *learned* professions. And among the means of this elevation, I think the one proposed, is of no small importance.

Surely, I hope the time is not far distant, when it will no longer be said, that only *one dentist in five* in our boasted New England, is a subscriber to a professional magazine.

